# Metabolic Profile, Antioxidant, Antimicrobial, Contractile, and Anti-Inflammatory Potential of *Moringa oleifera* Leaves (India)

**DOI:** 10.3390/life15040583

**Published:** 2025-04-01

**Authors:** Natalina Panova, Anelia Gerasimova, Yulian Tumbarski, Ivan Ivanov, Mina Todorova, Ivayla Dincheva, Galia Gentscheva, Vera Gledacheva, Valeri Slavchev, Iliyana Stefanova, Nadezhda Petkova, Stoyanka Nikolova, Krastena Nikolova

**Affiliations:** 1 Department of Physics and Biophysics, Faculty of Pharmacy, Medical University of Varna, 84 Tzar Osvbod-Tel, 9000 Varna, Bulgaria; panova@mu-varna.bg; 2 Department of Chemistry, Faculty of Pharmacy, Medical University of Varna, 84 Tzar Osvoboditel, 9000 Varna, Bulgaria; anelia.gerasimova@mu-varna.bg; 3 Department of Microbiology and Biotechnology, Technological Faculty, University of Food Technologies, 26 Maritsa Blvd., 4002 Plovdiv, Bulgaria; tumbarski@abv.bg; 4 Department of Organic Chemistry and Inorganic Chemistry, University of Food Technologies, 26 Maritsa Blvd., 4002 Plovdiv, Bulgaria; ivanov_ivan.1979@yahoo.com (I.I.); nadezhda_petkova@uft-plovdiv.bg (N.P.); 5 Department of Organic Chemistry, Faculty of Chemistry, University of Plovdiv, 4000 Plovdiv, Bulgaria; minatodorova@uni-plovdiv.bg (M.T.); tanya@uni-plovdiv.bg (S.N.); 6 Department of Agrobiotechnologies, AgroBioInstitute, Agricultural Academy, 8 Dragan Tsankov Blvd., 1164 Sofia, Bulgaria; ivadincheva@yahoo.com; 7 Department of Chemistry and Biochemistry, Medical University-Pleven, 1 Saint Kliment Ohridski Street, 5800 Pleven, Bulgaria; 8 Department of Medical Physics and Biophysics, Faculty of Pharmacy, Medical University of Plovdiv, 4002 Plovdiv, Bulgaria; vera.gledacheva@mu-plovdiv.bg (V.G.); valeri.slavchev@mu-plovdiv.bg (V.S.); iliyana.stefanova@mu-plovdiv.bg (I.S.)

**Keywords:** *Moringa oleifera*, metabolic profile, antioxidant activity, antibacterial activity, anti-inflammatory activity, spasmolytic activity

## Abstract

Background: *Moringa oleifera* is one of the most famous plants used for medicinal purposes. Its leaf extracts have antimicrobial activity and antioxidant activities, and reduce swelling in ulcers. Objectives: The present article aimed to determine the metabolic profile of Moringa oleifera leaf extracts from two samples originating from India and to evaluate in vitro and ex vivo their biological activities. Methods: The antioxidant properties of *Moringa oleifera* leaf extracts (methanol, 50% ethanol, and 70% ethanol) were studied in vitro for antimicrobial, anti-inflammatory, and antioxidant activities. The ex vivo contractile effects of the extracts were determined by assaying circularly isolated smooth muscle (SM) strips from a rat’s stomach. Results: The obtained results indicated that one of the samples had amino acid and organic acid content approximately twice that of the second sample. In all the tests, the 50% ethanol extract of both samples showed better antioxidant activity (209 mM TE 100 g^−1^ for the DPPH method). The 70% ethanolic extract of Sample 1 exhibited the strongest antimicrobial activity, inhibiting Gram-positive *Bacillus cereus*, *Bacillus subtilis*, and *Staphylococcus aureus*. The 50% ethanolic extract of both samples exhibited the highest anti-inflammatory activity, demonstrating a twice better effect than the reference drug diclofenac. Finally, the pre-incubation of SM preparations with both samples significantly enhanced the ACh-induced contractile response, increasing it by 134% and 111%, respectively. Conclusions: The findings suggested potential applications of *Moringa oleifera* as a suitable candidate for antimicrobial, antioxidant, anti-inflammatory, and antispasmodic supplementation for alternative pharmaceutical and nutraceutical products.

## 1. Introduction

*Moringa oleifera* is the most widely distributed species among the 13 in the family *Moringaceae* [1]. *M. oleifera* is a broad-leaved tree thriving in tropical and subtropical climates on slightly acidic to alkaline soils, with temperatures ranging from 25 °C to 35 °C [2,3,4]. The plant is distributed across Asia, Africa, the Caribbean, Latin America, the Pacific Islands, Florida, Madagascar, Central America, Cuba, the Philippines, Ethiopia, and Nigeria [5,6]. Due to its medicinal properties, it is cultivated in countries such as Mexico, Hawaii, South America, etc. It exhibits variations in chemical composition caused by substantial differences in soil and climatic conditions.

During the last decades, the limit of microbial diseases and infections has been exceeded drastically due to the increasing occurrence of resistance to antibiotics [7,8]. The proliferation of different types of microorganisms in the oral and dental regions is the cause of dental infections. These include the root canal system’s odontogenic infections and Periapical periodontitis, which are brought on by anaerobic bacteria such as *Candida albicans*, *Enteroccus faecalis*, and *Porphyromonas gingivalis* [9]. The development of novel antimicrobial drugs and herbal remedies is essential for the management of harmful bacteria, particularly for the treatment of diseases brought on by resistant bacteria. Therefore, medicinal plants possessing antibacterial properties can be considered as a potential source for the development of new antibacterial agents [7,10].

The pharmacological properties of *Moringa oleifera* are associated with all parts of the plant—roots, leaves, seeds, stem-bark, root-bark, and pods. Extracts from the leaves have antimicrobial activity [11], antioxidant activities [12], and reduce swelling in ulcers [13]. Traditional uses of the plant’s bark include treating toothache [14], hypertension [15], and paralysis [16]. People recognize extracts from the flowers as powerful aphrodisiacs, and they are known to alleviate conditions like peptic ulcers and splenomegaly [5]. Even though all the parts of the plant have biological activities, the leaves have better antimicrobial activity.

It is known that differences in climatic factors, soil composition, and growing conditions can significantly affect the content of bioactive compounds and biological properties of extracts [17,18], which are very important in the formulation of food supplements, cosmetics, or pharmaceutical products.

Therefore, the present article aims

- To determine the metabolic profile of *Moringa oleifera* leaf extracts from two samples from India;
- To evaluate in vitro their antioxidant, antimicrobial, anti-inflammatory, and ex vivo spasmolytic activity.

The obtained results may reveal new possibilities for the application of *Moringa oleifera* in new formulations as food supplements and pharmaceutical products.

## 2. Materials and Methods

### 2.1. Plant Material

Two samples of *Moringa oleifera* leaves were analyzed. The first one originated from India, the second one was purchased in Bulgaria. This study aims to compare the two samples and to identify both possible similarities and differences in the chemical content and biological activities of the samples.

Sample 1

The first sample consisted of dried leaves purchased from a farm located in the Western Ghats, Coimbatore district, Tamil Nadu, South India, with GPS coordinates 11.0168° N, 76.9558° E.

The drying process took place in well-ventilated rooms out of direct sunlight at a controlled temperature of 40 °C. The final moisture content was less than 10%, which was below the 12% level required by the pharmacopeia [19]. In laboratory conditions, the dried leaves were finely ground using a laboratory homogenizer (Bosch, Stuttgart, Germany). The powder was subsequently sieved through a 0.5 mm mesh sieve and stored in dry plastic containers until analysis.

Sample 2

The second sample is a *Moringa oleifera* leaf powder produced in India and purchased from a biomarket in Sofia, Bulgaria. The label identifies the sample’s origin (*Moringa oleifera* leaf powder, Origin: India. Manufacturer: Megasmart Bulgaria EOOD, Sofia, Bulgaria).

### 2.2. Test Microorganisms

To evaluate the antimicrobial activity of the extracts from *Moringa oleifera*, a set of microorganisms was used, provided by the Department of Microbiology at the University of Food Technologies (UFT), Plovdiv (Table 1).

*B. subtilis* and *B. cereus* were cultivated on Luria-Bertani agar supplemented with glucose (LBG agar) at 30 °C for 24 h, whereas *S. aureus*, *L. monocytogenes*, *E. faecalis*, *S. enteritidis*, *K. pneumoniae*, *E. coli*, *P. vulgaris*, and *P. aeruginosa* were cultivated on LBG agar at 37 °C for 24 h. *C. albicans* was cultivated on Malt Extract Agar (MEA) at 37 °C, whereas *S. cerevisiae* was cultivated on MEA at 30 °C for 24 h. The fungi *A. niger*, *A. flavus*, *P. chrysogenum*, and *F. moniliforme* were cultivated on MEA at 30 °C for 7 days or until sporulation.

### 2.3. Culture Media

Luria-Bertani agar supplemented with glucose (LBG agar) (Laboratorios Conda S.A., Madrid, Spain) was used for the cultivation of bacteria. Notably, 50 g of LBG agar medium base was dissolved in 1 L of deionized water at pH 7.5 ± 0.2. Malt extract agar (MEA) (Scharlab S.L., Madrid, Spain) was used for the cultivation of yeasts and fungi. Notably, 48 g of MEA medium base was dissolved in 1 L of deionized water (pH 5.6 ± 0.2). Both culture media were prepared according to the manufacturers’ instructions and autoclaved at 121 °C for 20 min before use.

### 2.4. Methods

#### 2.4.1. GC-MS Analysis

The sample preparation for the extraction of polar metabolites (organic and amino acids, sugars, and sugar alcohols) and lipids (fatty acids and sterols) using GC-MS is according to the methodology described by I. Dincheva [20].

Methanol extracts for determinations of organic and amino acids, sugars, fatty acids, and sterols were prepared by homogenizing samples using an Ul-tra-Turrax T25 Homogenizer (IKA, Staufen, Germany) at 10,000 rpm for 30 s. The homogenized samples were incubated in a TS-100 thermoshaker (Analytik Jena AG, Jena, Germany) at 120 rpm and 4 °C for 12 h. Following incubation, the samples were centrifuged using a centrifuge (Beckman Coulter, Brea, CA, USA) at 22 °C and 13,000 rpm for 10 min. The resulting supernatant was collected and used for chromatographic analysis.

GC-MS analysis was carried out using a 7890 A gas chromatograph (Agilent Technologies, Santa Clara, CA, USA), linked to a 5975C mass spectral detector (Agilent Technologies). The HP-5 ms capillary column had a length of 30 m, a diameter of 0.32 mm, and a film thickness of 0.25 µm silica, covered with a 0.25 µm film of dimethylsiloxane (Agilent) as a stationary phase. The oven temperature program was initial temperature 60 °C, hold 0 min, increase to 300 °C at 5 °C min^−1^, hold 10 min. The carrier gas flow rate (helium) was maintained at 1.0 mL min^−1^. The injector and detector temperatures were maintained at 250 °C. The mass detector scan range was 50 to 550 *m*/*z*. Injection volumes of 1 µL of the samples were performed in a 10:1 split mode. The mass spectra and metabolite identification were analyzed using the 2.73 AMDIS software (Automated Mass Spectral Deconvolution and Identification System, NIST, Gaithersburg, MD, USA). The separated compounds were matched against GC–MS spectra and retention indices (RI) of reference compounds available in the Golm Metabolome Database [21] and the NIST’08 database [22]. The software recorded the compounds’ RIs using a standard n-hydrocarbon calibration mixture (C_8_–C_36_, Restek, Teknokroma, Barcelona, Spain). Each kernel variety was analyzed in triplicate.

#### 2.4.2. LC-ESI-QTOF-MS

Extracts for determinations of flavonoids were prepared by homogenizing samples in 50% (*v*/*v*) ethanol using an Ultra-Turrax T25 Homogenizer (IKA, Staufen, Germany) at 10,000 rpm for 30 s. The homogenized samples were incubated in a TS-100 thermoshaker (Analytik Jena AG, Jena, Germany) at 120 rpm and 4 °C for 12 h. Following incubation, the samples were centrifuged using a centrifuge (Beckman Coulter, Brea, CA, USA) at 22 °C and 13,000 rpm for 10 min. The resulting supernatant was collected and used for chromatographic analysis.

The qualitative analysis of phenolic compounds from ethanolic extracts was carried out using an Agilent 1260 Infinity HPLC system coupled with an Agilent 6546 Accurate-Mass QTOF LC/MS system equipped with a Dual Agilent Jet Stream ESI source (Agilent Technologies, Santa Clara, CA, USA) and connected to a nitrogen generator (Parker Hannifin Corporation, Haverhill, MA, USA) providing nitrogen at a purity >99%. Samples were dissolved in water containing 0.1% formic acid, and 2 µL of the solution was injected into a Kinetex 2.6 µm C18 100 Å column (150 × 2.1 mm; Phenomenex, Torrance, CA, USA). The mobile phase consisted of phase A (water with 0.1% formic acid) and phase B (acetonitrile with 0.1% formic acid), with the following gradient: 0 min, 10% B; 1 min, 10% B; 15 min, 30% B; 22 min, 50% B; 28 min, 100% B; 34 min, 100% B; and 36 min, 10% B. The flow rate was set to 0.3 mL·min^−1^. The ESI-QTOF-MS analysis was performed in negative ionization mode with a 2 GHz extended dynamic range. Operating conditions included a fragmentor voltage of 160 V, drying gas temperature of 350 °C, a flow rate of 12 L·min^−1^, sheath gas temperature of 400 °C, a flow rate of 12 L·min^−1^, nebulizer pressure of 35 psi, capillary voltage of +4000 V, and skimmer voltage of 65 V. Data acquisition was performed in auto MS/MS mode with a mass range of 50–1000 amu for both MS and MS/MS experiments, at an acquisition rate of 1 spectrum per second. Collision-induced dissociation (CID) energy was optimized between 10 and 40 V. Data acquisition and analysis were carried out using Agilent’s Mass Hunter Data Acquisition Software (version B.03.01). The compound identification was carried out by comparing MS/MS spectra with literature data and the METLIN database.

#### 2.4.3. Quantification of Polyphenols via HPLC-PDA Analysis

The quantification of phenolic compounds in the samples was conducted using an Agilent 1260 series HPLC system (Agilent Technologies, CA, USA) equipped with a photodiode array (PDA) detector. The same column and operating conditions as described previously for LC-ESI-QTOF/MS were used, except the sample injection volume was set to 10 µL. The extracts were analyzed at wavelengths of 280 nm, 320 nm, and 370 nm through the PDA detector. Individual polyphenols were quantified by linear regression of external standard calibration curves, plotting peak area against concentration. Data acquisition and analysis were conducted using the Agilent LC-ESI-QTOF/MS MassHunter workstation software (Qualitative Analysis, version B.03.01, Agilent).

#### 2.4.4. Antioxidant Activity Assessment

DPPH radical scavenging capacity. The methanol extract (0.15 mL) was combined with 2.85 mL of a fresh 0.1 mM methanol solution of DPPH and measured as previously outlined [23].

ABTS radical scavenging capacity. 2.85 mL of freshly produced ABTS solution was combined with 0.15 mL of methanol extracts. Following a 15-min incubation at 37 °C in the absence of light, the absorbance was recorded at 734 nm [23].

The FRAP assay was conducted by combining 3.0 mL of FRAP reagent with 0.1 mL of extract, followed by a 10-min incubation at 37 °C in darkness, following which the absorbance was measured at 593 nm [23].

CuPRAC assay. The methanol extract (0.1 mL) was combined with 1 mL of 10 mM CuCl_2_, 1 mL of 7.5 mM methanol solution of Neocuproine, 1 mL of 0.1 M ammonium acetate buffer, and 1 mL of distilled H_2_O. Following a 20-min incubation at 50 °C, the absorbance was assessed at 450 nm. All antioxidant activity studies were conducted in triplicate and expressed as mM Trolox equivalents (mM TE) per 100 g extract [23].

#### 2.4.5. Total Phenolic Contents (TPC) and Total Flavonoid Contents (TFC)

The TPC was examined according to the methodology of Kujala et al. [24] with certain changes. Each extract (0.1 mL) was combined with 0.5 mL of Folin-Ciocalteu reagent and 0.4 mL of 7.5% Na_2_CO_3_. The mixture was vortexed and incubated for 5 min at 50 °C. Following incubation, the absorbance was measured at 765 nm. The total phenolic content (TPC) is quantified as milligrams of gallic acid equivalents (GAEs) per 100 g extract.

The total flavonoid content was assessed using the methodology outlined by Kivrak et al. [25]. A 0.5 mL aliquot of the material was combined with 0.1 mL of 10% Al(NO_3_)_3_, 0.1 mL of 1 M CH_3_COOK, and 3.8 mL of ethanol. Following a 40-min incubation at room temperature, the absorbance was measured at 415 nm. Quercetin (QE) served as a standard, and the results are shown as mg of quercetin equivalents (QE) per 100 g of extract.

#### 2.4.6. Antimicrobial Activity Assay

Extracts were obtained after maceration of 1 g of ground dried leaves of *Moringa oleifera* with 10 mL of methanol, 50% or 70% ethanol (Merck, Darmstadt, Germany). Samples were vortexed (IKA, model Vortex 3, Staufen, Germany) for 30 s and left in the dark at room temperature for 72 h. The resulting extracts were filtered through filter paper and stored at 4 °C. Before use, methanol and ethanol were evaporated under a vacuum, after which dissolved in pure DMSO (Dimethyl sulfoxide) liquid to 10 mg/mL concentration.

The antimicrobial activity of the extracts was determined by the agar well diffusion method [26]. After allowing the inoculated agar media to harden at room temperature, six wells (d = 6 mm) per Petri plate were made and duplicates of 60 μL of the extracts were pipetted into the agar wells. The plates were incubated under identical conditions.

The antibiotics Ampicillin and Penicillin (against bacteria), and Nystatin and Fluconazole (against yeast and fungi) in a concentration of 10 mg·mL^−1^ were used as controls.

The antimicrobial activity was determined by measuring the diameter of the inhibition zones around the wells on the 24th and 48th hour of incubation. Test microorganisms with an inhibition zone of 18 mm or more were considered sensitive; moderately sensitive were those in which the inhibition zone was from 12 to 18 mm; resistant were those in which the inhibition zone was up to 12 mm or completely missing.

In-depth biological evaluation included evaluation of their anti-inflammatory potential and antispasmodic activity.

#### 2.4.7. Inhibition of Albumin Denaturation

According to modern understanding, inflammation is a process that happens in reaction to a disruption or illness. The ability to stop inflammation is responsible for the anti-inflammatory properties of medication or therapy. Anti-inflammatory drugs reduce pain by lowering inflammation. This study aimed to assess the *Moringa oleifera* leaves potential in order to prevent albumin denaturation. In vitro, analysis of anti-inflammatory activity was assessed as inhibition of albumin denaturation estimating the degree of denaturation resistance of the albumin molecule. The method was carried out as described by Milusheva et al. [27,28]. Initially prepared methanol or ethanol *Moringa oleifera* leaf extracts were evaporated due to spontaneous denaturation of albumin in methanol or ethanol. Subsequently, 10 mg of the dry residue was dissolved in pure DMSO liquid to achieve a final solution concentration of 10 mg/mL.

The samples were prepared from 0.5 mL of a 5% aqueous solution of human albumin (Albunorm 20, Octapharma (IP) SPRL, 1070 Anderlecht, Belgium) and 0.2 mL of the diluted in DMSO tested *Moringa oleifera* extracts. The samples were incubated at 37 °C for 15 min. Each tube was filled with 2.5 mL of phosphate-buffered saline (pH 6.3), heated for 30 min to 80 °C, and then cooled for 5 min. The turbidity of the samples was measured spectrophotometrically at 660 nm (Cary 60 UV-Vis, Agilent Technologies, Santa Clara, CA, USA). A mixture of 2.5 mL of buffer and 0.2 mL of DMSO was used for the blank, while the product control contained 0.5 mL of serum albumin and 2.5 mL of buffer.

The percentage inhibition of protein denaturation was calculated according to the Formula (1):

(1) $$\mathrm{Percentage}\mathrm{of}\mathrm{inhibition}\mathrm{denaturation}=\frac{\left(Absorbance\;control-Absorbance\;sample\right)}{Absorbance\;control}\times 100$$

The control represents 100% protein denaturation. Commercially available anti-inflammatory drugs (diclofenac and acetylsalicylic acid) were used for comparison. Their anti-inflammatory effect was determined as a percentage of inhibition of albumin denaturation, following the same protocol as for the novel compounds.

### 2.5. In Vitro Experiments on Gastric SM Preparations from Rat Wistar

One gram of every sample was submerged in 20 mL of distilled water and sonicated at 30 °C for 1 h. The resultant leaf infusion was centrifuged for 5 min and subsequently filtered off using Whatman paper (Merck KGaA, Darmstadt, Germany).

Male Wistar rats with body weight in the range 240–280 g were used and reared with access to food and water, a 12 h light and 12 h dark cycle at 22 °C ± 1 °C, and humidity of 43%. The total number of rats used in the experiments was 10. Three or four muscle strips were taken from one rat’s stomach with the mucosal layer not violated. The number of muscle strips used for each data point was indicated by n. SM preparations of circular dissection, 12–13 mm in length and 1.0–1.1 mm in width were used to isometrically record the contractile activity (CA).

All the experiments were conducted under the requirements of the International Council for Ethical Guidelines for Animal Breeding Labs for Researchers, ARRIVE. All efforts were made to minimize the number of animals used and their suffering.

#### Method of Studying Spontaneous CA of Isolated SM Preparations

The SM strips were placed in a tissue bath containing Krebs solution (15 mL) and attached to a four-channel interface system to register the spontaneous CA of SM. All transducers used in our experiments were made by RODNOTY, Dublin, Ireland. The value of the initial mechanical tension of the preparations, obtained by stretching the tension system, corresponded to a tension force of 10 mN. To stabilize muscle tonus and spontaneous CA, approximately 60 min were allowed to elapse, during which period the Krebs solution in the tissue bath was changed 4 times. The baseline tonus value after this adaptation period was accepted as normal muscle activity. The drug-induced alterations (contraction or relaxation) were recorded as a positive or negative change in this value by Slavchev et al. [29]. SM tissue vitality was tested by adding 10^−6^ M Acetylcholine (ACh) at the beginning of the study and at the end of each application of the substances. ACh causes a contraction with significant force, and the magnitude of this force of contraction is taken as 100%. The forces of contraction caused by the other tested compounds were compared to the force caused by ACh. The results were presented in percentages (%).

### 2.6. Statistical Analysis

The SPSS 23.0 software (SPSS Inc., Chicago, IL, USA) was used for statistical analysis. All data were expressed as mean ± SD (standard deviation). Small values of the mean concentration of certain chemical compounds can lead to a higher standard deviation. Duncan’s test for multiple comparisons was performed to determine statistically significant differences between the two samples, as well as between extracts for anti-inflammatory and antioxidant activity. Statistical significance between two independent groups was analyzed by independent sample *t*-test. The level of statistical significance was considered as *p* < 0.05.

## 3. Results

### 3.1. Chemical Composition of the Leaves from Moringa oleifera

Table 2 presents the determination of flavonoids, phenolic acids, organic acids, and amino acids in the leaves of *Moringa oleifera* using GC-MS analysis. The comparisons made using the Duncan test for most components in Table 2, Table 3 and Table 4 showed statistically significant differences between the two samples.

The content of fatty acids and sterols in the samples is presented in Table 3.

The content of sugars is presented in Table 4.

### 3.2. Antioxidant Activity

The data on AOA for 50% and 70% ethanolic extracts and methanolic extract from *Moringa oleifera* leaves are presented in Table 5.

### 3.3. Antimicrobial Activity Results

The results from the antimicrobial activity test are shown in Table 6.

Methanolic and ethanolic extracts of *Moringa oleifera* leaves were tested for their antimicrobial activity against gram-positive and gram-negative bacteria, yeasts, and fungi. As seen from the results presented in Table 6, the methanolic extract of sample 1 exhibited low antimicrobial activity, expressed only against *P. aeruginosa*. The 50% and 70% ethanolic extracts of sample 1 inhibited the growth of *B. subtilis*, *B. cereus*, *S. aureus*, *L. monocytogenes*, *E. faecalis*, *S. enteritidis*, *E. coli*, and *P. aeruginosa*. The methanolic extract of sample 2 exhibited low antimicrobial activity, expressed against *B. subtilis*, *B. cereus*, *E. coli,* and *P. aeruginosa*. The 50% ethanolic extract of sample 2 inhibited the growth of *B. subtilis*, *B. cereus*, *L. monocytogenes*, *E. faecalis*, *S. enteritidis*, *E. coli*, and *P. aeruginosa*, whereas the 70% ethanolic extract showed stronger antimicrobial effect against the same test microorganisms and *S. aureus.* None of the extracts demonstrated antifungal activity.

### 3.4. Anti-Inflammatory Activity

**Figure 1:**
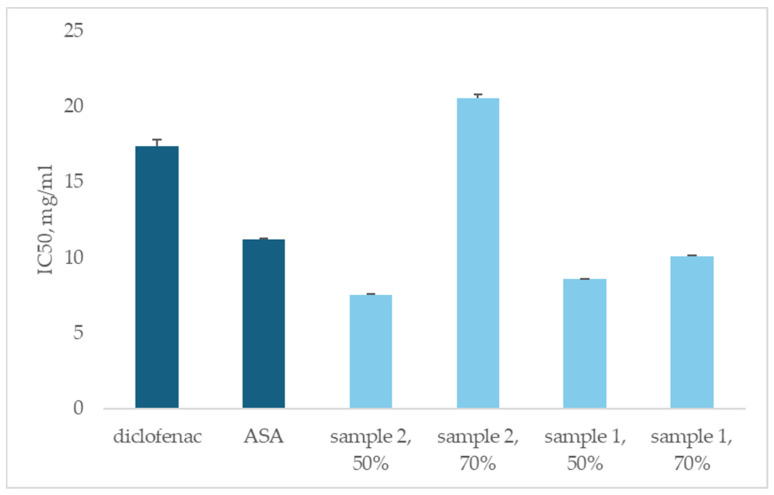
The inhibition of albumin denaturation activity of the *Moringa oleifera* extracts, compared to known anti-inflammatory drugs diclofenac and acetylsalicylic acid.

In vitro, the anti-inflammatory activity assay was evaluated as an inhibition of albumin denaturation, assessing the degree of resistance to denaturation of the human albumin molecule in the presence of plant extracts. The human albumin anti-denaturation method was used to evaluate the anti-inflammatory properties of the four *Moringa oleifera* extracts (Figure 1). Diclofenac and acetylsalicylic acid (ASA) are used as standard. The results were expressed as IC_50_.

### 3.5. Ex Vivo Experiments on Gastric SMs

**Figure 2:**
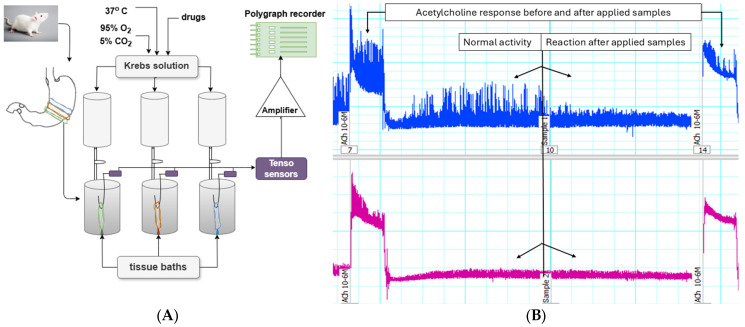
(**A**) Schematic representation of the experimental setup used to record the mechanical activity of circular gastric SM strips from rats in tissue baths. (**B**) Representative tracings illustrating the effects of *Moringa oleifera* leaves on the spontaneous CA of SM strips.

The effects of aqueous extracts of *Moringa oleifera* leaves on SM spontaneous CA are presented in Figure 2A,B and in Table 7.

## 4. Discussion

People frequently refer to *Moringa oleifera* as a “superfood” because of its rich chemical composition and physiological properties [30]. Both leaf samples analyzed in this study contain myristic, palmitic, arachidonic, and oleic acids, which share structural similarities with 10-hydroxy-2-decenoic acid, a compound found in royal jelly and honey [31]. Sample 1 exhibits a higher concentration of margaric acid (11.10 mg g^−1^) and myristic acid (5.30 mg g^−1^), whereas Sample 2 is richer in oleic acid (3.65 mg g^−1^). Izuta et al. stated that these fatty acids increase the production of Transforming Growth Factor-1 (TGF^−1^) and Vascular Endothelial Growth Factor (VEGF), which helps fibroblasts grow. This makes *Moringa* leaves ideal for use in wound healing [32].

Myristic acid, owing to its antimicrobial and anti-inflammatory properties, can help reduce skin inflammation, making the Indian *Moringa* leaves beneficial for treating skin infections. Meanwhile, the unsaturated oleic acid has cardioprotective potential, as it reduces LDL cholesterol and increases HDL cholesterol. Both samples also contain behenic acid in concentrations ranging from 4.67 to 6.17 mg g^−1^. While not an essential fatty acid, behenic acid is highly valued in cosmetic and food technologies. It soothes and alleviates dryness and sensitivity in the skin [33]. It can also serve as a solidifying agent in margarine production without requiring hydrogenation [33].

The fatty acid profiles of the Indian samples differ from those observed in other regions of the world. For example, linoleic acid content in Nigerian samples ranges between 1.5 and 2.0 mg g^−1^, which is comparable to the findings for Indian once. Guevara et al. [34] found that Cuban *Moringa oleifera* had a different fatty acid profile than Indian samples. The Cuban samples had less oleic acid and more palmitic acid. Al-Owaisi et al. [35] reported that linoleic acid content from Ethiopian samples at approximately 1.2 mg g^−1^, which is lower than the Indian samples.

The total phenolic acid and total flavonoid content were investigated exclusively for ethanol and methanol extracts of *Moringa oleifera* leaves. Extracts in hexane, petroleum ether, and other solvents were not prepared, as the study aimed at evaluating extracts for inclusion in dietary supplements. The samples with the highest amounts of total phenolic and flavonoid were extracted with 50% ethanol. This shows that there are polar phenolic compounds that can dissolve in both water and alcohol. The results obtained in this study correlate well with those reported by other authors [36].

The total phenolic content in our study for the 50% ethanol extract was 19.61 mg GAE/100 g and 23.06 mg GAE/100 g, which is a lot more than what Ahmed [18] reported for ethanol extracts from leaves, which was 3.09 mg GAE/100 g. However, the total flavonol content is lower than reported in the same study (540.07 µg g^−1^). Leone et al. found that an 80% ethanol extract had a total phenolic content of 8.21 µg GAE/100 g. This is much lower than the 20–22 mg GAE/100 g that we saw in our study [17].

In our study, *Moringa oleifera* leaves are rich in both essential and non-essential amino acids. Among the essential amino acids, phenylalanine (2.44 mg g^−1^ in Sample 1, 1.44 mg g^−1^ in Sample 2) and isoleucine (1.83 mg g^−1^ in Sample 2) are the most abundant. Out of the non-essential amino acids, most of the aspartic acid (6.21 mg g^−1^ in Sample 1 and 5.53 mg g^−1^ in Sample 2) and glutamic acid (4.77 mg g^−1^ in Sample 1 and 4.15 mg g^−1^ in Sample 2) were found. The sample obtained as dried leaves directly from a farm in India showed higher amino acid content, compared to the sample from Bulgaria. Technological processing or geographical differences may be the reason for this.

In samples from Nigeria, essential amino acids such as valine (1.2 mg g^−1^) and isoleucine (0.9 mg g^−1^) were found, which are comparable to the Indian samples [37]. However, studies from Ethiopia report lower levels of leucine (0.5 mg g^−1^) and valine (0.7 mg g^−1^) [17]. Analyses of leaves from cultivated *Moringa oleifera* in Pakistan revealed higher levels of essential amino acids [38]. This confirms the hypothesis that both the fatty acid and amino acid profiles vary significantly depending on climate, soil, and humidity.

The antioxidant power of ethanol and methanol extracts is linked to phenolic acids [39]. We obtained data using four methods: DPPH, ABTS, FRAP, and CUPRAC. In all of the tests, the 50% ethanol extract of both samples showed the most antioxidant activity (209 mM TE 100 g^−1^ for the DPPH method). The antioxidant potential in 50% and 70% ethanol extracts of the first sample determined by ABTS, FRAP, and CUPRAC methods had significantly higher activity compared to the second sample (five times, four times, and two times, respectively). The high antioxidant activity of the first sample is due to the higher concentrations of caffeic acid, chlorogenic acid, hyperoside, and isoquercitrin identified and quantified in the samples (Table 2). Samples from Ethiopia demonstrated similar DPPH activity of 200 mM Trolox/100 g in an 80% ethanol extract, comparable to our investigated samples from India. However, FRAP activity was significantly lower (25 mM Trolox 100 g^−1^) compared to the first sample, indicating a weaker capacity for iron ion reduction [35]. According to Leone [17], the samples from Ethiopia had ABTS activity of 85 mM Trolox 100 g^−1^ and DPPH activity of 170 mM Trolox 100 g^−1^. This is lower than Sample 1 but higher than Sample 2. The extracts’ high antioxidant activity can help protect the liver from damage caused by oxidative stress [40] and may also help protect against neurological diseases like Alzheimer’s and multiple sclerosis.

The agar diffusion technique was used to examine the antibacterial activity of the extracts on five Gram-positive, five Gram-negative, two yeasts, and four fungi. The good diffusion method reveals the level of sensitivity in pathogenic microorganisms; therefore, an organism susceptible to a chemical will not develop near the well in which it was placed. The zone of inhibition, also known as the clear zone, is the area that does not grow. The inhibition of the tested substance is proportional to the size of the clear zone.

The mean zones of inhibition in mm produced on the pathogenic microorganisms containing *M. oleifera* leaf extracts are presented in Table 6.

We found that both 70% *M. oleifera* leaf extracts were moderately sensitive against all the tested Gram-positive *Bacillus subtilis*, *Bacillus cereus*, *Staphylococcus aureus*, *Listeria monocytogenes*, *Enterococcus faecalis*, while both 50% are sensitive only against *Bacillus subtilis*, *Bacillus cereus*. For Gram-negative strains, both 70% and 50% extract of sample 1 were moderately sensitive against *Salmonella enteritidis* and *Escherichia coli*. This can be attributed to the difference in cell membrane permeability between the two bacterial strains, which is significantly affected by complex factors, such as the lipophobicity of the cell membrane, the thickness of the membrane and its surrounding layers, and the chemical composition of the antimicrobial drug [41].

Anzano et al. [42] previously investigated the antimicrobial activity of polar and apolar extracts from leaves and seeds of *M. oleifera* against two Gram-positive (*Staphylococcus aureus* and *Staphylococcus epidermidis*) and two Gram-negative (*Pseudomonas aeruginosa* and *Salmonella enterica*) bacteria and determined that both types of extracts obtained from leaves and seeds did not significantly inhibit the growth of the Gram-negative bacteria tested.

Another goal of this research is to evaluate the ability of *Moringa oleifera* extracts to prevent albumin denaturation. Figure 1 presents the results as half maximal inhibitory concentration (IC_50_). The 50% ethanolic extract (sample 2) exhibited the highest anti-inflammatory activity, followed by the 50% and 70% ethanolic extracts (sample 1). Seventy percent ethanol extract of *Moringa oleifera* (sample 2) exhibited the lowest anti-inflammatory activity.

Protein denaturation indicates the presence of an inflammatory process, during which the secondary and tertiary structures of proteins are destroyed, leading to the disruption of their biological functions. The ability of a substance or therapy to reduce inflammation is associated with an anti-inflammatory effect [43].

Non-steroidal and steroidal anti-inflammatory drugs reduce inflammation. However, these drugs have many adverse side effects. Anti-inflammatory drugs have shown a dose-dependent ability to inhibit thermally induced protein denaturation [27]. In our investigations, the plant extracts were compared to two anti-inflammatory drugs, diclofenac and ASA. The results showed that IC_50_ of 50% ethanolic extracts from samples, namely sample 2 (7.54 ± 0.06 mg mL^−1^) and sample 1 (8.61 ± 0.02 mg mL^−1^) exhibited 1.5 times better anti-inflammatory activity than ASA (11.20 ± 0.11 mg mL^−1^), resp. 2.5 times better anti-inflammatory activity than diclofenac (17.40 ± 0.40 mg mL^−1^). The activity of 70 % ethanolic extracts of sample 1 (10.08 ± 0.06 mg mL^−1^) was comparable to that of ASA and twice better than diclofenac. Seventy percent of the ethanolic extracts in sample 2 (20.58 ± 0.21 mg mL^−1^) had lower anti-inflammatory activity than the two controls.

Finally, we can conclude that 50% of both plant extracts of sample 1 and sample 2, as well as 70% of sample 1 had better albumin protection than the controls (Figure 1).

In addition to the antimicrobial and anti-inflammatory activities, the ex vivo spasmolytic potential of *Moringa oleifera* leaves has also been conducted. Previously, Caceres [44] investigated the antispasmodic potential of *Moringa oleifera* from Guatemala and found that only seeds have lower activity. Bearing in mind the fact that differences in climatic factors, soil composition, and growing conditions can significantly affect the biological activities of extracts [17,18], we aimed to evaluate the antispasmodic potential of *M. oleifera* leaves obtained from India.

SM tissues are typically bathed in a physiological solution (e.g., Krebs solution) that mimics body fluids. In experiments conducted with SM cells, the presence of ethanol or methanol is not permissible. Both alcohols directly affect CA. The alcohol is toxic to living cells and can alter muscle function by affecting ion channels, neurotransmitter release, and membrane integrity. Alcohol can denature proteins, dehydrate tissues, and disrupt normal physiological functions. Any recorded change in CA would be compromised and misinterpreted. Water-based extracts and infusions are commonly used in experiments to avoid these issues. By using water as a solvent, researchers can preserve the physiological properties of SM tissues and ensure that observed effects on CA are due to the test compounds rather than solvent interference. Additionally, water extracts better mimic natural biological conditions, making them a preferred choice for pharmacological and physiological studies on SM function.

The influence of the contractile function of the smooth muscles (SMs) comprising the gastrointestinal tract and vascular walls is pivotal in physiology and pathophysiology. Consequently, identifying compounds that affect the mechanisms of smooth muscle contraction and relaxation is of significant interest. A critical technique for this purpose is measuring SMs contraction or relaxation of native tissues by studying them under tissue bath conditions (ex vivo). Moreover, it is important to note that this type of research is both applicable and informative for monitoring endogenous and exogenous effects. To date, *Moringa* extracts have been regarded as biologically active substances but have not been investigated as effectors of the spontaneous rhythmic activity of SMs responsible for peristalsis.

The primary mechanokinetic parameters (tonus, amplitude, and frequency) were assessed using circularly isolated SM strips from the corpus part of the rat stomach. From the supernatants obtained using the method mentioned above, varying volumes of 5 to 150 µL were applied to the tissue baths to construct dose-response curves. Within the tested range, the sample volumes that demonstrated the most substantial impact were between 20 and 30 µL. As a result, for all subsequent experiments, a volume of 30 µL was chosen as the optimal parameter to ensure maximum effectiveness.

The observed contractile changes in tonus and amplitude of contractions under the influence of *Moringa oleifera* leaf extracts suggest differential involvement of the extracts in Ca^2+^ homeostasis, which is primarily responsible for the spontaneous CA of SMs. Their initial action induces a mild tonic relaxation response without a measurable change in the frequency of single contractions. Both extracts caused a significant reduction in the amplitude of spontaneous contractile reactions, with the inhibitory effect being more pronounced in Sample 1.

It is well established that contraction and relaxation processes in SMs are initiated by changes in free ionized Ca^2+^ levels in the extracellular or intracellular environment [45]. Moringa extracts likely influenced these Ca^2+^ concentrations, thereby modulating spontaneous CA. This hypothesis is further supported by results showing variations in SM responses when the substances are applied synergistically with one of the primary neurotransmitters ACh. Pre-incubation of SM preparations with the two tested substances significantly enhanced the ACh-induced contractile response. These findings suggest that the observed effects may primarily be related to the positive modulation of Ca^2+^ influx, a critical factor for spontaneous gastric SM contractions [46,47].

## 5. Conclusions

The determined metabolic profile of the leaves samples from *Moringa oleifera* shows both similarities and differences, although they originate from the same species, for example, the content of hyperoside, quinic acid, margaric acid, behenic acid, cerotic acid and β-sitosterol are different. The best antioxidant activity is possessed by 50% alcohol solutions for the first sample. Our results demonstrated that 70% ethanol extracts gave good results against all Gram-positive bacterial strains and two Gram-negative. Both 50% plant extracts and 70% extract of sample 1 had better albumin protection than the controls diclofenac and ASA. Therefore, the extract can be used as a natural anti-inflammatory agent. Studies evaluating the contractile potential of aqueous solutions of *Moringa oleifera* leaves showed that pre-incubation of SM preparations significantly enhanced the ACh-induced contractile response, increasing it by 134% and 111%, respectively. Therefore, *Moringa oleifera* has the potential to regulate gastrointestinal motility and could be considered for use in functional disorders such as irritable bowel syndrome.

Based on its high antioxidant, antimicrobial, and anti-inflammatory activities, *Moringa oleifera* extracts can be incorporated into dietary supplements aimed at patients with chronic inflammatory diseases or as alternatives to antimicrobial agents. For this purpose, further research on the synergy between *Moringa oleifera* and traditional antibiotics is necessary.

The *Moringa oleifera* Sample 1 purchased from a farm located in the Western Ghats, Coimbatore district, Tamil Nadu, South India shows higher antioxidant, anti-inflammatory, and spasmolytic activity due to its higher content of phenolic acids and flavonoids compared to the sample 2.

## Figures and Tables

**Table 1:** Test microorganisms used in the study.

Bacteria		Fungi	Yeasts
Gram-Positive	Gram-Negative		
*Bacillus subtilis* ATCC 6633	*Salmonella enteritidis* ATCC 13076	*Aspergillus niger* ATCC 1015	*Candida albicans* NBIMCC 74
*Staphylococcus aureus* ATCC 25923	*Klebsiella pneumoniae* ATCC 13883	*Aspergillus flavus*	*Saccharomyces cerevisiae* ATCC 9763
*Bacillus cereus* NCTC 11145	*Escherichia coli* ATCC 25922	*Penicillium chrysogenum*	
*Listeria monocytogenes* NBIMCC 8632	*Proteus vulgaris* ATCC 6380	*Fusarium moniliforme* ATCC 3893	
*Enterococcus faecalis* ATCC 29212	*Pseudomonas aeruginosa* ATCC 9027		

**Table 2:** Content of flavonoids, phenolic, organic, and amino acids in *Moringa oleifera*.

	Leaves Sample 1	Leaves Sample 2
	Content ± SD, mg g^−1^ Extract	Content ± SD, mg g^−1^ Extract
**Amino acids**		
**Non-essential amino acid**		
l-Glutamic acid	4.77 ± 0.11 ^a^	4.15 ± 0.10 ^b^
**Essential amino acid**		
l-Valine	1.00 ± 0.23 ^a^	0.44 ± 0.10 ^b^
l-Leucine	0.37 ± 0.09 ^b^	0.73 ± 0.17 ^a^
l-Isoleucine	0.93 ± 0.22 ^b^	1.83 ± 0.43 ^a^
Phenylalanine	2.44 ± 0.57 ^a^	1.44 ± 0.34 ^b^
l-Threonine	1.20 ± 0.28 ^a^	1.07 ± 0.25 ^a,b^
**Conditional non-essential amino acid**		
l-Aspartic acid	6.21 ± 1.45 ^a^	5.53 ± 1.29 ^b^
Proline	1.24 ± 0.29 ^a^	n.d.
Pyroglutamic acid	1.72 ± 0.40 ^a^	0.66 ± 0.15 ^b^
l-Serine	0.62 ± 0.15 ^a^	0.50 ± 0.12 ^a,b^
**Organic acids**		
Malic acid	3.92 ± 0.92 ^a^	3.45 ± 0.81 ^a,b^
Succinic acid	0.87 ± 0.20 ^a,b^	1.19 ± 0.28 ^a^
Fumaric acid	0.50 ± 0.12 ^b^	0.94 ± 0.22 ^a^
Citric acid	0.33 ± 0.11 ^b^	0.51 ± 0.14 ^a^
Succinic acid	0.25 ± 0.08 ^a^	0.33 ± 0.16 ^a^
Quinic acid	6.64 ± 0.77 ^a^	3.01 ± 0.45 ^b^
**Phenolic acids**		
Gentisic acid	0.67 ± 0.16 ^a^	0.40 ± 0.12 ^b^
Neochlorogenic acid	0.85 ± 0.23 ^a^	0.58 ± 0.17 ^b^
Chlorogenic acid	3.26 ± 0.90 ^a^	1.80 ± 0.48 ^b^
Caffeic acid	1.09 ± 0.29 ^a^	0.38 ± 0.19 ^b^
3-p-Coumaroylquinic acid	0.78 ± 0.30 ^a^	0.62 ± 0.15 ^a,b^
ρ-Coumaric acid	0.41 ± 0.08 ^a^	0.28 ± 0.07 ^a,b^
**Flavonoids**		
Hyperoside (Quercetin 3-galactoside)	6.74 ± 0.83 ^a^	4.59 ± 0.71 ^b^
Isoquercitrin (Quercetin 3-glucoside)	1.99 ± 0.29 ^a^	1.05 ± 0.18 ^b^
Quercetin 3-(6″-malonylglucoside)	1.24 ± 0.46 ^a^	0.64 ± 0.16 ^b^
Rutin (Quercetin 3-rutinoside)	2.85 ± 0.82 ^a^	2.00 ± 0.67 ^a,b^
Quercetin 3-(6″-acetylglucoside)	0.96 ± 0.15 ^a^	0.71 ± 0.22 ^a,b^
Kaempherol-3-*O*-α-rhamnoside	0.57 ± 0.30 ^a^	0.30 ± 0.11 ^b^
Kaempferol 3-galactoside	1.48 ± 0.21 ^a^	1.18 ± 0.24 ^a,b^
Kaempferol-3-*O*-glucoside	1.19 ± 0.37 ^a^	0.70 ± 0.29 ^b^
Kaempferol-3-*O*-rutinoside	0.76 ± 0.29 ^a^	0.63 ± 0.28 ^a,b^

Means in a row with a common superscript letter (a,b) differ (*p* < 0.05) as analyzed by Duncan test.

**Table 3:** Content of fatty acids and sterols in the leaves of *Moringa oleifera*.

	Leaves Sample 1	Leaves Sample 2
	Content ± SD, mg g^−1^ Extract	Content ± SD, mg g^−1^ Extract
**Fatty acids**		
Miristic acid (C14:0)	5.30 ± 0.72 ^a^	4.36 ± 0.59 ^b^
Oleic acid (C18:1)	0.56 ± 0.07 ^b^	3.65 ± 0.49 ^a^
Palmitic acid (C16:0)	3.24 ± 0.44 ^a,b^	3.96 ± 0.53 ^a^
Margaric acid (C17:0)	11.10 ± 1.50 ^a^	2.37 ± 0.32 ^b^
Linoleic acid (C18:2)	1.74 ± 0.23 ^a^	1.07 ± 0.14 ^b^
Stearic acid (C18:0)	1.04 ±0.14 ^b^	2.13 ± 0.29 ^a^
Arachidic acid (C20:0)	1.47 ± 0.20 ^b^	2.38 ± 0.32 ^a^
Behenic acid (C22:0)	4.67 ± 0.63 ^b^	6.17 ± 0.83 ^a^
Lignoceric acid (C24:0)	5.20 ± 0.70 ^a^	1.31 ± 0.18 ^b^
Cerotic acid (C26:0)	8.32 ± 1.12 ^a^	2.26 ± 0.30 ^b^
**Sterols**		
β-Amyrin	1.48 ± 0.20 ^a^	0.80 ± 0.11 ^b^
α-Amyrin	0.70 ± 0.09 ^b^	1.15 ± 0.16 ^a^
Betulin	0.82 ± 0.11 ^b^	2.26 ± 0.31 ^a^
β-Sitosterol	4.99 ± 0.67 ^b^	7.10 ± 0.96 ^a^
Stigmasterol	3.29 ± 0.44 ^a^	2.48 ± 0.33 ^b^

Means in a row with a common superscript letter (a,b) differ (*p* < 0.05) as analyzed by Duncan test.

**Table 4:** Mono- and disaccharide content in *Moringa oleifera* leaves.

	Leaves Sample 1	Leaves Sample 2
	Content ± SD, mg g^−1^ Extract	Content ± SD, mg g^−1^ Extract
**Saccharides (mono-, di-)**		
Fructose	15.72 ± 3.68 ^b^	19.95 ± 4.67 ^a^
Glucose	12.09 ± 2.83 ^b^	16.54 ± 3.87 ^a^
Sucrose	2.44 ± 0.46 ^b^	3.13 ± 0.73 ^a^
**Sugar alcohols**		
Myo-Inositol	30.00 ± 7.02 ^a^	26.25 ± 6.14 ^b^
Sorbitol	12.90 ± 3.02 ^a^	10.88 ± 2.55 ^b^

Means in a row with a common superscript letter (a,b) differ (*p* < 0.05) as analyzed by Duncan test.

**Table 5:** Antioxidant activity, total phenolic content, and total flavonoid content in extracts from leaves of *Moringa oleifera*.

Sample	TPC, mg GAE 100 g^−1^	TFC, μg QE 100 g^−1^	Antioxidant Activities, mM TE 100 g^−1^			
			DPPH	ABTS	FRAP	CuPRAC
*Moringa oleifera* leaves (CH_3_OH), sample 1	11.49 ± 0.15 ^e^	26.83 ± 1.60 ^f^	142.91 ± 5.14 ^d^	52.55 ± 0.58 ^c^	29.46 ± 2.00 ^b^	80.49 ± 7.36 ^c^
*Moringa oleifera* leaves (50% C_2_H_5_OH), sample 1	23.06 ± 0.11 ^a^	87.73 ± 0.50 ^a^	208.90 ± 9.10 ^a^	105.46 ± 3.60 ^a^	44.67 ± 2.50 ^a^	164.37 ± 8.28 ^b^
*Moringa oleifera* leaves (70% C_2_H_5_OH), sample 1	22.10 ± 0.14 ^b^	83.76 ± 1.10 ^b^	209.46 ± 5.14 ^a^	92.03 ± 0.29 ^b^	46.26 ± 1.75 ^a^	170.22 ± 7.36 ^a^
*Moringa oleifera leaves* (CH_3_OH), sample 2	14.08 ± 0.08 ^d^	44.79 ± 2.50 ^e^	108.51 ± 2.37 ^e^	18.93 ± 0.60 ^f^	7.56 ± 0.03 ^d^	50.36 ± 0.10 ^e^
*Moringa oleifera leaves* (50% C_2_H_5_OH), sample 2	19.61 ± 0.12 ^c^	77.83 ± 4.03 ^c^	193.52 ± 3.07 ^b^	27.76 ± 0.80 ^d^	14.34 ± 0.06 ^c^	83.35 ± 0.09 ^c^
*Moringa oleifera leaves* (70% C_2_H_5_OH), sample 2	19.59 ± 0.11 ^c^	63.78 ± 1.40 ^d^	155.49 ± 0.79 ^c^	23.97 ± 0.57 ^e^	12.62 ± 0.04 ^c^	63.58 ± 0.06 ^d^

Means in a column with a common superscript letter (a–f) differ (*p* < 0.05) as analyzed by Duncan test.

**Table 6:** Antimicrobial activity of *Moringa oleifera* leaf extracts.

Test Microorganism	Inhibition Zones, mm									
	Leaf Extracts (10 mg mL^−1^) Sample 1			Leaf Extracts (10 mg mL^−1^) Sample 2			Controls * (10 mg mL^−1^)			
	CH_3_OH	50% C_2_H_5_OH	70% C_2_H_5_OH	CH_3_OH	50% C_2_H_5_OH	70% C_2_H_5_OH	A	P	N	F
*Bacillus subtilis* ATCC 6633	-	16.0 ± 0.0	17.0 ± 0.0	8.0 ± 0.0	12.5 ± 0.7	16.0 ± 0.0	16.0 ± 0.0	-	n.a.	n.a.
*Bacillus cereus* NCTC 11145	-	14.5 ± 0.7	17.0 ± 0.0	9.0 ± 0.0	13.0 ± 0.0	16.0 ± 0.0	20.0 ± 0.0	-	n.a.	n.a.
*Staphylococcus aureus* ATCC 25923	-	11.0 ± 0.0	13.0 ± 0.0	-	-	13.0 ± 0.0	35.0 ± 0.0	30.0 ± 0.0	n.a.	n.a.
*Listeria monocytogenes* NBIMCC 8632	-	11.0 ± 0.0	12.5 ± 0.7	-	9.5 ± 0.7	12.0 ± 0.0	40.0 ± 0.0	12.0 ± 0.	n.a.	n.a.
*Enterococcus faecalis* ATCC 29212	-	12.0 ± 0.0	13.0 ± 0.0	-	10.0 ± 0.0	12.5 ± 0.7	38.0 ± 0.0	-	n.a.	n.a.
*Salmonella enteritidis* ATCC 13076	-	12.0 ± 0.0	13.0 ± 0.0	-	10.0 ± 0.0	12.5 ± 0.7	40.0 ± 0.0	-	n.a.	n.a.
*Klebsiella pneumoniae* ATCC 13883	-	-	-	-	-	-	25.0 ± 0.0	-	n.a.	n.a.
*Escherichia coli* ATCC 25922	-	13.0 ± 0.0	14.0 ± 0.0	9.5 ± 0.7	11.0 ± 0.0	14.0 ± 0.0	16.0 ± 0.0	-	n.a.	n.a.
*Proteus vulgaris* ATCC 6380	-	-	-	-	-	-	30.0 ± 0.0	-	n.a.	n.a.
*Pseudomonas aeruginosa* ATCC 9027	8.0 ± 0.0	11.0 ± 0.0	13.0 ± 0.0	10.0 ± 0.0	11.0 ± 0.0	14.0 ± 0.0	16.0 ± 0.0	-	n.a.	n.a.
*Candida albicans* NBIMCC 74	-	-	-	-	-	-	n.a.	n.a.	22.0 ± 0.0	-
*Saccharomyces cerevisiae* ATCC 9763	-	-	-	-	-	-	n.a.	n.a.	31.0 ± 0.0	-
*Aspergillus niger* ATCC 1015	-	-	-	-	-	-	n.a.	n.a.	32.0 ± 0.0	25.0 ± 0.0
*Aspergillus flavus*	-	-	-	-	-	-	n.a.	n.a.	26.0 ± 0.0	20.0 ± 0.0
*Penicillium chrysogenum*	-	-	-	-	-	-	n.a.	n.a.	26.0 ± 0.0	13.0 ± 0.0
*Fusarium moniliforme* ATCC 38932	-	-	-	-	-	-	n.a.	n.a.	25.0 ± 0.0	-

* Controls: A—Ampicillin; P—Penicillin; N—Nystatin; F—Fluconazole; n.a.—no activity.

**Table 7:** Changes in the muscle tonus, amplitude and frequency of the contractile response of SM under the influence of *Moringa oleifera* leaves, as well as their effects on the ACh-induced response.

Applied Substance	Tonus, mN	Amplitude, mN	Frequency, Number/min	Time for Reaction, min	ACh-Induced SMresponse, %	n
Normal activity	2.00 ± 0.10	2.40 ± 0.18	5.00 ± 0.05	20.00 ± 0.20	100%	10
*Moringa oleifera* Sample 1	1.88 ± 0.13	1.02 ± 0.11 ^a^	5.10 ± 0.15	13.50 ± 0.30	134%	9
*Moringa oleifera* Sample 2	1.90 ± 0.12	1.67 ± 0.15 ^a^	5.0. ± 0.12	15.00 ± 0.20	111%	8

The comparison is between the spontaneous CA in Krebs solution before and after applying the tested substances. All data are presented as mean ± SEM. The number of SM strips used in each experiment was indicated by n. Statistically significant differences are marked with ^a^ (*p* < 0.05).

## Data Availability

Datasets from the time of this study are available from the respective authors upon reasonable request.
